# Ligand-dependent spatiotemporal signaling profiles of the μ-opioid receptor are controlled by distinct protein-interaction networks

**DOI:** 10.1074/jbc.RA119.008685

**Published:** 2019-09-12

**Authors:** Srgjan Civciristov, Cheng Huang, Bonan Liu, Elsa A. Marquez, Arisbel B. Gondin, Ralf B. Schittenhelm, Andrew M. Ellisdon, Meritxell Canals, Michelle L. Halls

**Affiliations:** ‡ Drug Discovery Biology Theme, Monash Institute of Pharmaceutical Sciences, Monash University, Parkville 3052, Victoria, Australia; § Monash Proteomics and Metabolomics Facility, Monash University, Clayton 3800, Victoria, Australia; ¶ Monash Biomedicine Discovery Institute, Monash University, Clayton 3800, Victoria, Australia

**Keywords:** G protein-coupled receptor (GPCR), opiate opioid, cell compartmentalization, protein complex, proteomics, extracellular signal–regulated kinase (ERK), desmosome, Ras-related C3 botulinum toxin substrate 1 (Rac1), cell signaling, opioid-based analgesics

## Abstract

Ligand-dependent differences in the regulation and internalization of the μ-opioid receptor (MOR) have been linked to the severity of adverse effects that limit opiate use in pain management. MOR activation by morphine or [d-Ala^2^,*N*-MePhe^4^, Gly-ol]enkephalin (DAMGO) causes differences in spatiotemporal signaling dependent on MOR distribution at the plasma membrane. Morphine stimulation of MOR activates a Gα_i/o_–Gβγ–protein kinase C (PKC) α phosphorylation pathway that limits MOR distribution and is associated with a sustained increase in cytosolic extracellular signal-regulated kinase (ERK) activity. In contrast, DAMGO causes a redistribution of the MOR at the plasma membrane (before receptor internalization) that facilitates transient activation of cytosolic and nuclear ERK. Here, we used proximity biotinylation proteomics to dissect the different protein-interaction networks that underlie the spatiotemporal signaling of morphine and DAMGO. We found that DAMGO, but not morphine, activates Ras-related C3 botulinum toxin substrate 1 (Rac1). Both Rac1 and nuclear ERK activity depended on the scaffolding proteins IQ motif-containing GTPase-activating protein-1 (IQGAP1) and Crk-like (CRKL) protein. In contrast, morphine increased the proximity of the MOR to desmosomal proteins, which form specialized and highly-ordered membrane domains. Knockdown of two desmosomal proteins, junction plakoglobin or desmocolin-1, switched the morphine spatiotemporal signaling profile to mimic that of DAMGO, resulting in a transient increase in nuclear ERK activity. The identification of the MOR-interaction networks that control differential spatiotemporal signaling reported here is an important step toward understanding how signal compartmentalization contributes to opioid-induced responses, including anti-nociception and the development of tolerance and dependence.

## Introduction

The μ-opioid receptor (MOR)^3^ is a G protein–coupled receptor (GPCR) that mediates the effects of opioid-based analgesics such as morphine (1). Analgesics targeting the MOR remain the most effective drugs for the treatment of severe pain. This is despite limitations associated with chronic use of opioid analgesics, including the development of dependence, addiction, and respiratory depression (2), contributing factors to opioid-induced overdose deaths that have increased in the last decade (3).

The MOR is differentially regulated by morphine compared with other high-efficacy opioid ligands, such as the enkephalin analogue, [d-Ala^2^,*N*-MePhe^4^,Gly-ol]-enkephalin (DAMGO). In particular, morphine causes limited receptor phosphorylation and recruitment of the scaffolding protein β-arrestin, which results in compromised receptor internalization and resensitization (4–7). The distinct ligand-dependent regulatory profiles of MOR also correlate with differences in spatiotemporal signaling. Previously, we have shown that DAMGO causes a redistribution of the MOR at the plasma membrane, and this is associated with a transient increase in both cytosolic and nuclear extracellular signal-regulated kinase (ERK) that occurs prior to receptor internalization (8). In contrast, morphine is unable to cause any change in MOR distribution at the plasma membrane. This is due to activation of a Gα_i/o_–Gβγ–protein kinase C (PKC) α phosphorylation pathway, and it results in a sustained increase in cytosolic ERK only. Release of the morphine-stimulated MOR from this limited microdomain (by inhibition of Gβγ, PKC, or disruption of the lipid organization of the plasma membrane) caused redistribution of the receptor and activation of nuclear ERK (8). Therefore, different ligands cause clear differences in the spatial organization of the receptor that controls its spatiotemporal signaling. The molecular mechanism through which this change in receptor distribution leads to distinct signaling patterns remains to be addressed.

The activation of different spatiotemporal signaling pathways by a GPCR can be controlled by a change in receptor location (8, 9), but it may also be dependent on the organization of different effector/regulatory proteins in close proximity to the receptor (10, 11). Such a protein-interaction network could be physically associated with the GPCR or occur in close proximity within a localized area of the plasma membrane. Until recently, identification of a GPCR-signaling complex required prior knowledge of candidate proteins (*e.g.* functionally, by targeting likely candidates with chemical inhibitors or gene silencing, or physically, by choosing targets to identify during immunoblotting or immunostaining). Confirmation of a physical interaction within a complex could then be confirmed by biochemical or imaging techniques (*e.g.* co-immunoprecipitation, FRET, or proximity ligation assay). This approach has its limitations, as the investigator must first choose a likely candidate, and biochemical isolation of protein complexes usually preserves only very strong/direct interactions. The rise of proximity biotinylation proteomics approaches (12, 13) has provided an unbiased method to identify candidate proteins that may form protein-interaction networks with a GPCR and has the distinct advantage over affinity purification–based approaches of detecting transient, weak, and more distal interactions.

Here, we have used APEX2 (ascorbate peroxidase from the soy bean) proximity biotinylation proteomics to show that activation of MOR by DAMGO or morphine induces the assembly of different protein-interaction networks, and that these protein-interaction networks are critical for the activation of downstream signaling. DAMGO stimulation of the MOR led to increased proximity of the receptor to proteins that are essential for receptor internalization, but also identified protein networks involved in the activation of small G proteins. Based on this, we found a DAMGO-mediated activation of Rac1, a small G protein of the Rho family of GTPases that are important regulators of cytoskeletal organization and trafficking (14). The activation of Rac1 by MOR depended on two scaffolding proteins, IQ motif-containing GTPase-activating protein-1 (IQGAP1) and Crk-like protein (CRKL). Moreover, both IQGAP1 and CRKL were required for the DAMGO-mediated activation of nuclear ERK. In contrast, morphine stimulation of the MOR led to an increased proximity of the receptor to protein networks that are critical for the formation of desmosomes. Desmosomes are specialized regions of the plasma membrane that form cell–cell junction complexes and can assemble signaling hubs (15, 16). Knockdown of desmosomal proteins desmocolin-1 (DSC1) and junction plakoglobin (JUP) transformed the morphine spatiotemporal signaling profile into that of DAMGO: transient activation of nuclear ERK. This suggests that the restricted distribution of MOR in response to morphine (and therefore activation of sustained ERK within the cytoplasm) is, at least in part, dependent on the formation of desmosome protein networks around the receptor. These data show that the distinct spatiotemporal signaling profiles stimulated by DAMGO compared with morphine activation of the MOR are not only dependent on the spatial organization of the receptor at the plasma membrane but also on the assembly of distinct protein-interaction networks in close proximity to the receptor.

## Results

### MOR–APEX2 maintains the spatiotemporal ERK signaling of the WT MOR

**Figure 1.**
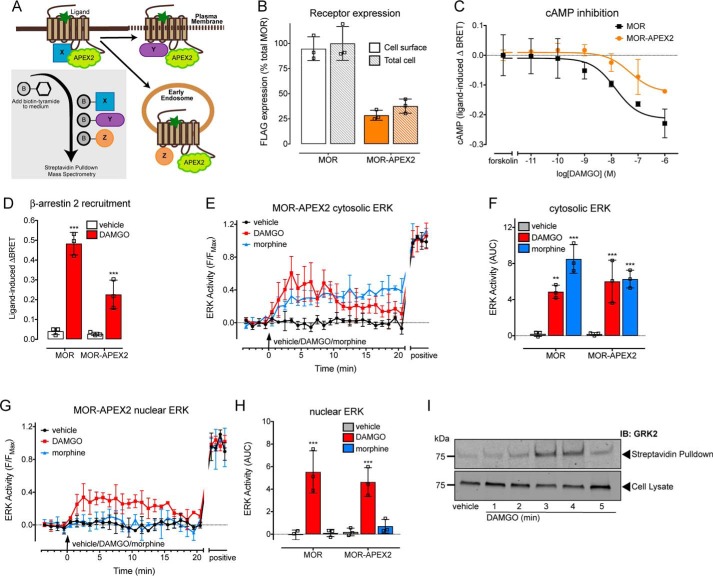
**Using APEX2 proximity biotinylation proteomics to identify novel MOR-interaction networks.** *A,* addition of an APEX2 tag to the C terminus of the MOR allows biotinylation of interacting and proximal proteins (within 20 nm) following addition of biotin-tyramide to the cell culture medium, and activation of the APEX2 enzyme by hydrogen peroxide. After the reaction is quenched, cells are lysed, and the biotinylated proteins are isolated by streptavidin pulldown and identified by MS. *B,* in-cell Western showing cell-surface expression of MOR WT (*MOR*) and APEX2-tagged (*MOR–APEX2*) transiently expressed in HEK293 cells (*n* = 3). *C,* inhibition of forskolin-stimulated cAMP following DAMGO stimulation of HEK293 cells transiently expressing MOR or MOR–APEX2 and a BRET biosensor for cAMP (*n* = 3). *D,* recruitment of β-arrestin 2-Venus to KRas-RLuc8 in HEK293 cells co-expressing GRK2 and MOR (control) or MOR–APEX2 in response to stimulation with 1 μm DAMGO was determined using BRET (*n* = 3). Data are expressed as the 20-min area under the curve. *E–H*, analysis of the spatial activation of ERK in HEK293 cells transiently expressing MOR or MOR–APEX2 and stimulated with vehicle, 10 nm DAMGO, or 100 nm morphine. *E*, analysis of cytosolic ERK activity using cytoEKAR (*n* = 3). *F,* AUC from *E* and Fig. S2A. *G*, analysis of nuclear ERK activity using nucEKAR (*n* = 3). *H,* AUC from *G* and Fig. S2*B*. **, *p* < 0.05; ***, *p* < 0.001 *versus* vehicle control, two-way ANOVA with Dunnett's multiple comparison test. *I,* analysis of the proximity of MOR–APEX2 to transiently expressed GRK2 in HEK293 cells following stimulation with 1 μm DAMGO, streptavidin pulldown, and immunoblotting. Scatter plots show individual data points; *symbols/bars* represent means, and *error bars* indicate standard deviation of the mean from *n* experiments as stated.

To identify protein-interaction networks that are important for activation of the distinct spatiotemporal signaling profiles elicited by DAMGO *versus* morphine stimulation of the MOR, we employed APEX2 (17) proximity biotinylation proteomics (Fig. 1*A*). The APEX2 tag was added to the C-terminal tail of the FLAG–MOR (herein referred to as MOR) to generate FLAG–MOR–APEX2 (herein referred to as MOR–APEX2). As demonstrated previously for the MOR transiently expressed in HEK293 cells (8), all MOR–APEX2 were expressed at the cell surface (Fig. 1*B* and Fig. S1*A*), inhibited forskolin-stimulated cAMP (Fig. 1*C*), and recruited β-arrestin 2 to the plasma membrane in response to DAMGO (Fig. 1*D*). The lower level of expression of MOR-APEX (∼31% compared with MOR) was reflected in the smaller degree of cAMP inhibition and β-arrestin 2 recruitment to the cell surface in response to DAMGO. Importantly, MOR–APEX2 also maintained the ligand-dependent spatiotemporal signaling profiles of MOR. Activation of both MOR and MOR–APEX2 with an EC_80_ concentration of DAMGO (10 nm) (8) caused a transient increase in cytosolic and nuclear ERK (Fig. 1, *E–H*, and Fig. S2). In contrast, an EC_80_ concentration of morphine (100 nm) (8) caused a sustained increase in cytosolic ERK, but no change in nuclear ERK activity compared with a vehicle control (Fig. 1, *E–H*, and Fig. S2). Therefore, MOR–APEX2 is functional and maintains the ligand-dependent spatiotemporal signaling profile of MOR.

### Activation of MOR–APEX2 by DAMGO causes biotinylation of G-protein receptor kinase (GRK)2

Following the addition of biotin-tyramide to the cell culture medium, and activation by hydrogen peroxide, APEX2 biotinylates competent proximal proteins with its activity decreasing with distance over a 20-nm radius (18, 19). The biotinylated proteins can be identified by cell lysis and streptavidin pulldown, followed by either immunoblotting or MS. To confirm functionality of the APEX2 tag within MOR–APEX2, we stimulated HEK293 cells transiently expressing MOR–APEX2 and a well-characterized regulatory protein GRK2 (7) with 1 μm DAMGO (Fig. 1*I*) or 1 μm morphine (Fig. S1*B*) over a time course of 5 min. GRK2 and GRK3 are important for receptor phosphorylation following activation by DAMGO, but not morphine (5, 7). As anticipated, the addition of DAMGO caused an increase in the biotinylation of GRK2 that peaked at 3–4 min, before declining at 5 min (Fig. 1*I* and Fig. S1*C*). In contrast, there was no change in the amount of biotinylated GRK2 in response to morphine (Fig. S1, *B* and *C*). Interestingly, the kinetics of GRK2 biotinylation occur over a longer time scale than our previous measurements of GRK2 recruitment using bioluminescence resonance energy transfer (BRET) and FRET (plateau at 2 min) (7). This suggests a lag period between the recruitment of the protein detected by BRET/FRET, and the ability to observe a detectable change in the biotinylation status of the same protein by immunoblotting.

### Proximity biotinylation after activation of MOR–APEX2

**Figure 2.**
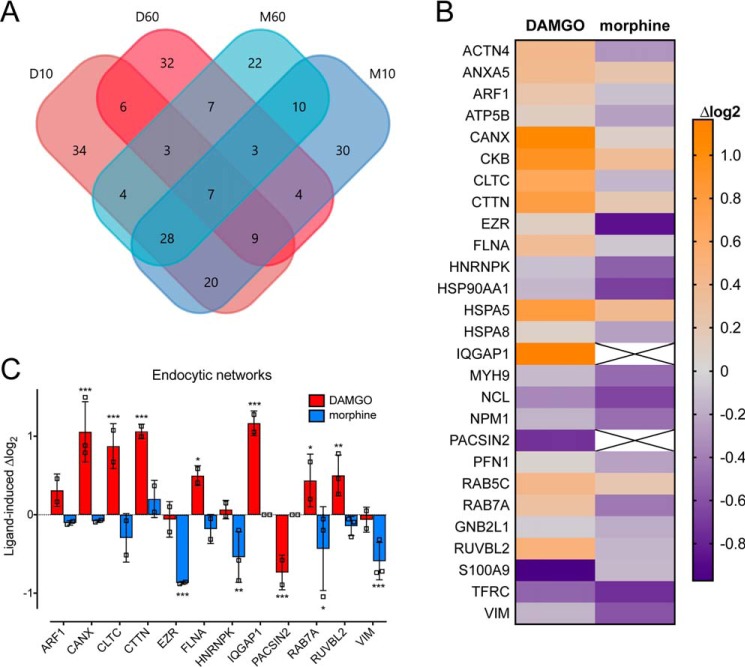
**Stimulation of MOR–APEX2 with DAMGO, but not morphine, identifies protein networks important for endocytosis.** *A,* stimulation of HEK293 cells transiently expressing MOR–APEX2 with vehicle, 1 μm DAMGO, or 1 μm morphine for 10 or 60 min identified 374 biotinylated proteins. The Venn diagrams indicate the number of proteins that were significantly enriched for each treatment condition (>1.5-fold change compared with vehicle control or present in stimulated samples but absent in vehicle-treated samples and vice versa), including the number of unique proteins and the number of proteins that were identified in multiple treatment conditions. *D10*, DAMGO 10 min; *D60*, DAMGO 60 min; *M60*, morphine 60 min; and *M10,* morphine 10 min. *B* and *C*, as expected, stimulation of MOR–APEX2 with DAMGO, but not morphine, enriched proteins that are important in endocytic networks, as determined by IPA software. *B,* heat map showing proteins from the endocytic network that were increased (*orange*) or decreased (*purple*) following stimulation of MOR–APEX2 with DAMGO or morphine for 60 min compared with a vehicle control. Data are expressed as the average log_2_ change in protein abundance compared with vehicle (*n* = 3). *C,* proteins from *B* that were differentially affected by DAMGO *versus* morphine treatment. Data are expressed as the log_2_ change in protein abundance compared with vehicle control, with scatter plots showing the individual data points; *bars* represent the mean, and *error bars* indicate the standard deviation of the mean of three independent experiments. *, *p* < 0.05; **, *p* < 0.01; and ***, *p* < 0.001 *versus* vehicle control, two-way ANOVA with Dunnett's multiple comparison test.

As MOR–APEX2 activates the same spatiotemporal signaling pathways as MOR, and we can detect time-dependent changes in the biotinylation of proteins important in receptor regulation, we performed APEX2 proximity biotinylation assays followed by tandem MS (LC-MS/MS) to identify other proteins likely to contribute to ligand-dependent signaling. Cells transiently expressing MOR–APEX2 were treated with vehicle, 1 μm DAMGO, or 1 μm morphine for 10 min (after receptor redistribution but prior to internalization) or 60 min (after receptor internalization) (8). We used saturating concentrations of DAMGO and morphine to ensure full receptor occupancy and therefore maximal receptor activation and biotinylation of proximal proteins. Importantly, differential spatiotemporal signaling of the MOR is maintained in response to 1 μm DAMGO and 1 μm morphine (Fig. 4*D*). APEX2 was activated by a 1-min treatment with hydrogen peroxide at the end of the stimulation period, before quenching, cell lysis, and isolation of biotinylated proteins by streptavidin pulldown. Biotinylated proteins from three biological replicates were identified and quantified by LC-MS/MS (Table S1). In total, 374 proteins were identified across three independent replicates of the six experimental conditions, with 219 proteins showing a change in enrichment of more than 1.5-fold compared with vehicle controls (Fig. 2*A*). Although some proteins were enriched across all treatment groups, we also identified proteins that were unique to both drug treatment and time of stimulation. In some cases, proteins were absent in vehicle-treated samples but present following stimulation with DAMGO or morphine, or vice versa (*i.e.* ligand-induced). If this occurred in all three independent replicates, we imputed a fixed value for the missing conditions to analyze the data (see “Experimental procedures”).

To validate the collected data set, we initially looked for protein-interaction networks relating to receptor endocytosis. This was chosen as it is well-documented that DAMGO causes robust internalization of the MOR by 60 min, whereas morphine causes very limited receptor internalization (8). Ingenuity pathway analysis (IPA) software identified a network of proteins important for endocytosis that was differentially affected by DAMGO *versus* morphine stimulation of MOR–APEX2 (Fig. 2, *B* and *C*, and Table 1). For example, stimulation of MOR–APEX2 for 60 min with DAMGO, but not morphine, caused an enrichment in proteins essential for endocytosis, including clathrin heavy chain (CLTC) and Rab7a.

**Table 1:** **Proteins from interaction networks important for endocytosis** The proteins and average log_2_ values are listed for the endocytic protein-interaction network shown as a heat map in Fig. 2*B*. Biotinylated proteins in HEK293 cells transiently expressing MOR–APEX2 were isolated and identified following stimulation with vehicle, DAMGO, or morphine for 60 min. Data are expressed as the log_2_ change compared with vehicle.

Gene name	Protein name	Log_2_ value	
		DAMGO	Morphine
*ACTN4*	Actinin α4	0.395	−0.296
*ANXA5*	Annexin A5	0.379	0.224
*ARF1*	ADP ribosylation factor 1	0.235	−0.104
*ATP5F1B*	ATP synthase F1 subunit β	0.113	−0.244
*CANX*	Calnexin.	1.056	0.091
*CKB*	Creatine kinase B	0.928	0.352
*CLTC*	Clathrin heavy chain	0.652	−0.146
*CTTN*	Cortactin	0.775	0.201
*EZR*	Ezrin	0.11	−0.868
*FLNA*	Filamin A	0.356	−0.069
*HNRNPK*	Heterogeneous nuclear ribonucleoprotein K	−0.102	−0.539
*HSP90AA1*	Heat-shock protein 90α family class A member 1	−0.147	−0.678
*HSPA5*	Heat-shock protein familyA (Hsp70) member 5	0.795	0.379
*IQGAP1*	IQ motif containing GTPase-activating protein 1	1.165	0
*MYH9*	Myosin heavy chain 9	−0.129	−0.489
*NCL*	Nucleolin	−0.359	−0.651
*NPM1*	Nucleophosmin 1	−0.159	−0.475
*PACSIN2*	Protein kinase C and casein kinase substrate in neurons 2	−0.735	0
*PFN1*	Profilin 1	0.054	−0.24
*RAB5C*	RAB5C, member RAS oncogene family	0.426	0.209
*RAB7A*	RAB7A, member RAS oncogene family	0.294	−0.432
*RACK1*	Receptor for activated C kinase 1	−0.049	−0.193
*RUVBL2*	RuvB like AAA ATPase 2	0.5	−0.144
*S100A9*	S100 calcium-binding protein A9	−0.972	−0.139
*TFRC*	Transferrin receptor	−0.52	−0.744
*VIM*	Vimentin	−0.161	−0.591

Interestingly, whereas stimulation of MOR–APEX2 for 10 min identified some proteins that were differentially affected by DAMGO *versus* morphine, IPA software did not identify any protein-interaction networks that were differentially affected by the two ligands at this time point (Fig. S3). In contrast, differences in protein-interaction networks were more apparent following a 60-min stimulation with ligand, so these interaction networks were examined in more detail.

### DAMGO activates Rac1 signaling, dependent on IQGAP1 and CRKL

**Figure 3.**
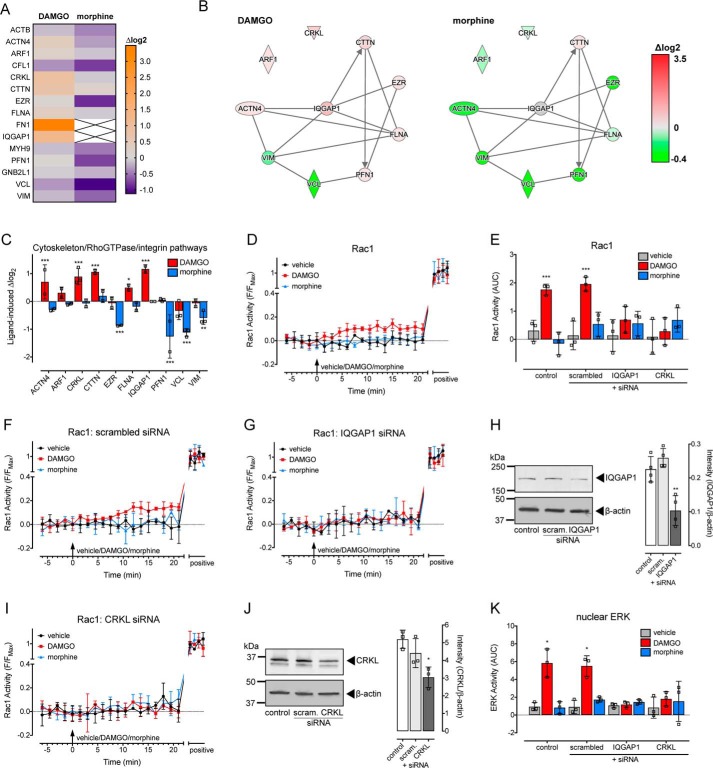
**DAMGO, but not morphine, activates Rac1 and its spatiotemporal signaling profile depends on the scaffolding proteins IQGAP1 and CRKL.** *A–C*, stimulation of MOR–APEX2 with DAMGO, but not morphine, for 60-min enriched proteins involved in actin cytoskeleton signaling, signaling by Rho family GTPases, and integrin signaling (*n* = 3). *A,* heat map showing proteins from these three pathways that were increased (*orange*) or decreased (*purple*) following treatment with DAMGO or morphine compared with a vehicle control. Data are expressed as the average log_2_ change in protein abundance compared with vehicle. *B,* interaction networks for the proteins that were differentially affected by DAMGO (*left panel*) *versus* morphine (*right panel*) were determined by IPA. *Red* indicates increased abundance, and *green* indicates a decrease. Data are expressed as the average log_2_ change in protein abundance compared with a vehicle control. *C,* proteins from *B*, with data expressed as the log_2_ change in protein abundance compared with vehicle control. *, *p* < 0.05; **, *p* < 0.01; and ***, *p* < 0.001 *versus* vehicle control, two-way ANOVA with Dunnett's multiple comparison test. *D–J*, analysis of the activation of Rac1 in single HEK293 cells transiently expressing MOR and RaichuEV–Rac1 and stimulated with vehicle, 1 μm DAMGO, or 1 μm morphine. *D,* time course of Rac1 activity (*n* = 3). *E,* AUC of data from *D, F, G,* and *I*. ***, *p* < 0.001 *versus* vehicle control, two-way ANOVA with Dunnett's multiple comparison test. *F,* time course of Rac1 activity in cells co-transfected with scrambled siRNA (*n* = 3). *G,* time course of Rac1 activity in cells co-transfected with IQGAP1 siRNA (*n* = 3). *H,* confirmation of knockdown of IQGAP1 protein by immunoblotting. *Left panel* is a representative blot, and *right panel* is the grouped intensity measurements from four independent experiments. **, *p* < 0.01 *versus* pcDNA transfected control, one-way ANOVA with Dunnett's multiple comparisons test. *I,* time course of Rac1 activity in cells co-transfected with CRKL siRNA (*n* = 3). *J,* confirmation of knockdown of CRKL protein by immunoblotting. *Left panel* is a representative blot, and *right panel* is the grouped intensity measurements from three independent experiments. *, *p* < 0.05 *versus* pcDNA transfected control, one-way ANOVA with Dunnett's multiple comparisons test. *K,* AUC of nuclear ERK measured in single HEK293 cells transiently expressing MOR and nucEKAR, and stimulated with vehicle, 1 μm DAMGO, or 1 μm morphine, under control conditions or following co-transfection with scrambled siRNA, IQGAP1 siRNA, or CRKL siRNA (*n* = 3). AUC was calculated from the time courses in Fig. S6. *, *p* < 0.05 *versus* vehicle control, two-way ANOVA with Dunnett's multiple comparison test. Scatter plots show individual data points; *symbols/bars* represent means, and *error bars* indicate standard deviation of the mean from *n* experiments as stated.

We first looked for novel protein-interaction networks that were specific for DAMGO stimulation of MOR–APEX2. Three interconnected canonical pathways were identified that were exclusively affected by DAMGO: (i) actin cytoskeleton signaling; (ii) signaling by Rho family GTPases; and (iii) integrin signaling. We listed all proteins within these three canonical pathways that were identified in at least two out of the three biological replicates in response to DAMGO. Of this list, if a protein was found in only one biological replicate for vehicle or morphine treatment, it was excluded due to low confidence. The only exception to this was when a protein was completely absent in vehicle-treated samples but present in two out of the three biological replicates following a drug treatment, or vice versa (*i.e.* ligand-dependent protein interactions). In this particular case and to visualize and analyze these data, missing values needed to be imputed. This stringent analysis criteria left a list of 15 proteins (Fig. 3*A* and Table 2), of which the abundance of 10 were differentially affected by DAMGO *versus* morphine treatment (Fig. 3, *B* and *C*).

**Table 2:** **Proteins from interaction networks important for DAMGO signaling** The proteins and average log_2_ values are listed for interaction networks important for actin cytoskeletal signaling, signaling by Rho family GTPases, and integrin signaling shown as a heat map in Fig. 3*A*. Biotinylated proteins in HEK293 cells transiently expressing MOR–APEX2 were isolated and identified following stimulation with vehicle, DAMGO or morphine for 60 min. Data are expressed as the log_2_ change compared with vehicle.

Gene name	Protein name	Log_2_ value	
		DAMGO	Morphine
*CFL1*	Cofilin 1	−0.366	−0.799
*VCL*	Vinculin	−0.335	−1.117
*VIM*	Vimentin	−0.161	−0.591
*ACTB*	Actin β	−0.134	−0.434
*MYH9*	Myosin heavy chain 9	−0.129	−0.489
*RACK1*	Receptor for activated C kinase 1	−0.049	−0.193
*PFN1*	Profilin 1	0.054	−0.764
*EZR*	Ezrin	0.11	−0.868
*ARF1*	ADP ribosylation factor 1	0.235	−0.104
*FLNA*	Filamin A	0.356	−0.069
*ACTN4*	Actinin α4	0.395	−0.296
*CTTN*	Cortactin	0.775	0.201
*CRKL*	CRK like proto-oncogene, adaptor protein	0.893	−0.068
*IQGAP1*	IQ motif–containing GTPase-activating protein 1	1.165	0
*FN1*	Fibronectin 1	3.494	0

IPA software highlighted signaling by Rho family GTPases (RhoA, Rac1, and Cdc42), and only DAMGO caused a large increase in the abundance of IQGAP1 (Fig. 3*C*), which binds activated Cdc42 and Rac1 (but not RhoA) (20). We therefore looked for activation of Rac1 and Cdc42 in single live cells using the FRET biosensors previously described (21, 22). We observed no effect of DAMGO or morphine on the activity of Cdc42 in HEK293 cells transiently expressing MOR and Raichu–Cdc42 FRET biosensor (Fig. S4). In contrast, DAMGO, but not morphine, stimulated a small but significant increase in Rac1 activity following MOR stimulation, as detected by the RaichuEV–Rac1 FRET biosensor (Fig. 3, *D* and *E*). We then determined whether IQGAP1 was important for the increase in Rac1 activity in response to DAMGO. There was no effect of a scrambled siRNA control on the Rac1 signal (Fig. 3, *E* and *F*); however, knockdown of IQGAP1 using targeted siRNA (mean ± S.D.: 54.07 ± 19.50% knockdown relative to control) abolished the Rac1 signal in response to DAMGO (Fig. 3, *E*, *G*, and *H*). In addition to IQGAP1, another protein with known scaffolding functions, CRKL, was uniquely enriched by DAMGO stimulation of MOR–APEX2 (Fig. 3*C*). CRKL was of particular interest as it has SH2/SH3 protein-interaction domains and is implicated in the activation of ERK signaling (23, 24). As observed for IQGAP1, knockdown of CRKL using targeted siRNA (mean ± S.D.: 41.38 ± 11.16% knockdown relative to control) abolished the DAMGO-stimulated increase in Rac1 activity (Fig. 3, *E*, *I*, and *J*). There was no effect of knockdown of either IQGAP1 or CRKL on endogenous levels of Rac1 (Fig. S5). Moreover, knockdown of both IQGAP1 and CRKL also abolished the DAMGO-stimulated increase in nuclear ERK activity (Fig. 3*K* and Fig. S6), as measured using the nucEKAR FRET biosensor (25). There was no effect of knockdown of IQGAP1 or CRKL on the maximal FRET change for either FRET biosensor (Fig. S7). It is interesting to note that while the potential role of IQGAP1 and CRKL was identified due to increased biotinylation after 60 min of MOR stimulation, knockdown of the two proteins abolished ERK signaling at a much earlier time point (5 min). This “time discrepancy” is likely due to the different levels of amplification and detection thresholds for each assay. So while biotinylation of proteins proximal to the MOR (unamplified) requires longer to reach the detection threshold of the assay, activation of downstream signaling (highly amplified) can be detected much earlier.

Therefore, activation of MOR by DAMGO, but not morphine, causes an increase in Rac1 activity that depends on the scaffolding proteins IQGAP1 and CRKL, and both IQGAP1 and CRKL are required for DAMGO-mediated activation of nuclear ERK.

### Spatiotemporal ERK-signaling profile of morphine is controlled by desmosomal proteins

**Figure 4.**
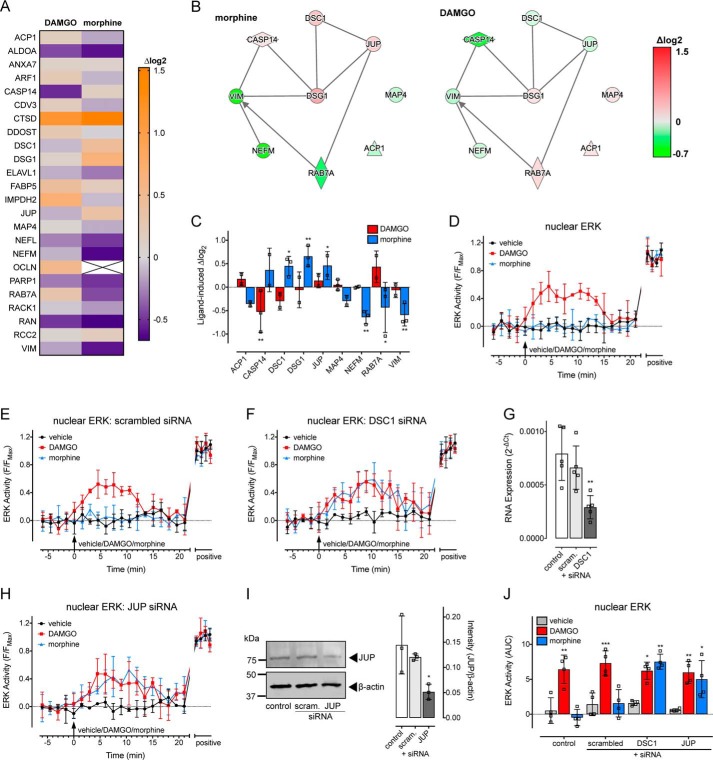
**Morphine, but not DAMGO, increases the proximity of MOR to desmosomal proteins, which control its spatiotemporal signaling profile.** *A–C*, stimulation of MOR–APEX2 with morphine, but not DAMGO, for 60-min enriched an interaction network that included desmosomal proteins (*n* = 3). *A,* heat map showing proteins from this network that were increased (*orange*) or decreased (*purple*) following treatment with 1 μm morphine or 1 μm DAMGO compared with a vehicle control. Data are expressed as the average log_2_ change in protein abundance compared with vehicle. *B,* interaction networks for the proteins that were differentially affected by morphine (*left panel*) *versus* DAMGO (*right panel*) were determined by IPA. *Red* indicates increased abundance, and *green* indicates a decrease. Data are expressed as the average log_2_ change in protein abundance compared with a vehicle control. *C,* proteins from *B*, with data expressed as the log_2_ change in protein abundance compared with vehicle control. *, *p* < 0.05; **, *p* < 0.01; and ***, *p* < 0.001 *versus* vehicle control, two-way ANOVA with Dunnett's multiple comparison test. *D–J,* analysis of the activation of nuclear ERK in single HEK293 cells transiently expressing MOR and nucEKAR, and stimulated with vehicle, 1 μm morphine, or 1 μm DAMGO. *D,* time course of nuclear ERK activity (*n* = 4). *E,* time course of nuclear ERK activity in cells co-transfected with scrambled siRNA (*n* = 4). *F,* time course of nuclear ERK activity in cells co-transfected with DSC1 siRNA (*n* = 4). *G,* confirmation of knockdown of DSC1 by quantitative RT-PCR (*n* = 5). **, *p* < 0.01 *versus* pcDNA control, one-way ANOVA with Dunnett's multiple comparisons test. *H,* time course of nuclear ERK activity in cells co-transfected with JUP siRNA (*n* = 4). *I,* confirmation of knockdown of JUP protein by immunoblotting. *Left panel* is a representative blot, and *right panel* is the grouped intensity measurements from three independent experiments. *, *p* < 0.05 *versus* pcDNA transfected control, one-way ANOVA with Dunnett's multiple comparisons test. *J,* AUC from *D–F* and *H*. *, *p* < 0.05; **, *p* < 0.01; ***, *p* < 0.001 *versus* vehicle control, two-way ANOVA with Dunnett's multiple comparison test. Scatter plots show individual data points, and *symbols/bars* represent means, and *error bars* indicate standard deviation of the mean from *n* experiments as stated.

We then looked for protein-interaction networks that were specific for morphine activation of MOR–APEX2. We identified a network of inter-related proteins that were uniquely affected by morphine. This list was filtered based on the same criteria used for the DAMGO-specific proteins (*i.e.* proteins have to be identified in at least two out of three biological replicates), to leave 24 proteins (Fig. 4*A* and Table 3), of which an abundance of nine were differentially affected by morphine *versus* DAMGO treatment (Fig. 4, *B* and *C*).

**Table 3:** **Proteins from interaction networks important for morphine signaling** The proteins and average log_2_ values are listed for interaction networks identified as important for morphine signaling shown as a heat map in Fig. 4*A*. Biotinylated proteins in HEK293 cells transiently expressing MOR–APEX2 were isolated and identified following stimulation with vehicle, DAMGO or morphine for 60 min. Data are expressed as the log_2_ change compared with vehicle.

Gene name	Protein name	Log_2_ value	
		DAMGO	Morphine
*ACP1*	Acid phosphatase 1	0.175	−0.154
*ALDOA*	Aldolase, fructose-bisphosphate A	−0.437	−0.596
*ANXA7*	Annexin A7	0.107	0.084
*ARF1*	ADP ribosylation factor 1	0.235	−0.104
*CASP14*	Caspase 14	−0.524	0.144
*CDV3*	CDV3 homolog	0.185	−0.149
*CTSD*	Cathepsin D	1.147	1.523
*DDOST*	Dolichyl-diphosphooligosaccharide–protein glycosyltransferase noncatalytic subunit	0.27	−0.017
*DSC1*	Desmocollin 1	−0.09	0.451
*DSG1*	Desmoglein 1	0.101	0.658
*ELAVL1*	ELAV-like RNA-binding protein 1	−0.126	−0.292
*FABP5*	Fatty acid-binding protein 5	0.418	0.192
*IMPDH2*	Inosine monophosphate dehydrogenase 2	0.694	−0.086
*JUP*	Junction plakoglobin	−0.119	0.352
*MAP4*	Microtubule-associated protein 4	0.049	−0.138
*NEFL*	Neurofilament light	−0.264	−0.484
*NEFM*	Neurofilament medium	−0.113	−0.641
*OCLN*	Occludin	0.527	0
*PARP1*	Poly(ADP-ribose) polymerase 1	−0.289	−0.498
*RAB7A*	RAB7A, member RAS oncogene family	0.294	−0.432
*RACK1*	Receptor for activated C kinase 1	−0.049	−0.193
*RAN*	RAN, member RAS oncogene family	−0.492	−0.667
*RCC2*	Regulator of chromosome condensation 2	0.057	0.181
*VIM*	Vimentin	−0.161	−0.591

Of these proteins, we identified an interaction-network between Rab7a, JUP, DSC1, DSG1, and caspase-14 (CASP14). JUP, DSC1, and DSG1 proteins are integral for the formation of desmosomes (15), and both CASP14 and Rab7a have been linked to these structures (26). Desmosomes are specialized and highly-ordered membrane domains that mediate cell–cell contact and strong adhesion (15). We have previously shown that the integrity of the plasma membrane is critical for the control of MOR spatiotemporal signaling and that morphine stimulation of MOR restricts the plasma membrane distribution of the receptor via a Gα_i/o_–Gβγ–PKCα pathway (8). Inhibition of the Gβγ–PKCα pathway, or disruption of the architecture of the plasma membrane using lipid disruptors, allowed morphine to redistribute the MOR and activate transient ERK signals in the cytosol and nucleus (8). Given that PKCα is necessary for desmosome formation (27, 28), we assessed whether the close proximity of the MOR to desmosome protein networks controlled the morphine-stimulated ERK spatiotemporal signaling profile.

DSC1 and DSG1 are expressed at low levels in HEK293 cells, and we were unable to detect expression at the protein level by immunoblotting (Fig. S8, *A* and *B*). Although we could detect DSC1 expression at the RNA level using quantitative PCR (Fig. 4*G*), we were unable to do so for DSG1. This suggests that DSG1 expression must be enriched in the vicinity of MOR in HEK293 cells such that we could detect biotinylated DSG1 using LC-MS/MS but not DSG1 at the protein/RNA level from whole cells. We therefore focused on the desmosomal proteins DSC1 and JUP. The proteins were knocked down using targeted siRNA, and we measured nuclear ERK activity using the nucEKAR FRET biosensor in HEK293 cells expressing MOR (Fig. 4, *D–J*). Knockdown of DSC1 (mean ± S.D.: 63.13 ± 13.73% knockdown relative to control) and JUP (mean ± S.D.: 64.32 ± 10.57% knockdown relative to control) by targeted siRNA was confirmed using quantitative RT-PCR (Fig. 4*G*) or immunoblotting (Fig. 4*I*), respectively. There was no effect of a scrambled siRNA control (Fig. 4, *E* and *J*); however, knockdown of DSC1 (Fig. 4, *F*, *G*, and *J*) or JUP (Fig. 4, *H–J*) allowed morphine to stimulate a transient increase in nuclear ERK. There was no effect of knockdown of these proteins on the ability of DAMGO to increase nuclear ERK (Fig. 4, *D–J*).

Therefore, morphine, but not DAMGO, activation of MOR leads to an increased proximity of the receptor to desmosome protein networks. Knockdown of desmosomal proteins DSC1 or JUP facilitates a transient increase in nuclear ERK in response to morphine.

## Discussion

**Figure 5.**
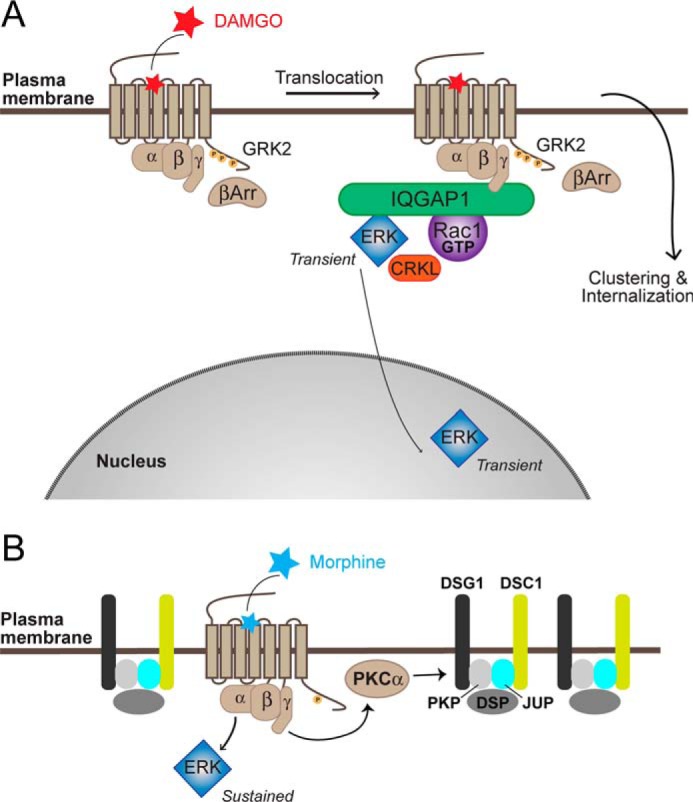
**DAMGO and morphine differentially assemble protein-interaction networks to control MOR spatiotemporal signaling.** *A,* DAMGO activation of the MOR leads to rapid recruitment of GRK2 and β-arrestins and facilitates a redistribution of the MOR at the plasma membrane (66). The DAMGO-stimulated receptor is in close proximity to IQGAP1, a large scaffolding protein that is required for the activation of Rac1 and nuclear ERK. IQGAP1 has been previously shown to bind both ERK and Rac1 (38). The DAMGO-stimulated MOR also associates with the adaptor protein, CRKL, which is essential for the activation of both Rac1 and nuclear ERK. The activation of Rac1 and nuclear ERK (10 min) occurs prior to receptor internalization (30–60 min (8)). *B,* in contrast, morphine activation of the MOR stimulates a Gα_i/o_–Gβγ–PKCα pathway, which increases the proximity of MOR to desmosomal proteins. Desmosomes are formed by two transmembrane proteins, DSC and DSG, which in turn interact with the intracellular proteins plakoglobin/JUP, plakophilin (*PKP*) and desmoplakin *(DSP*) (15). The formation of stabilized desmosomal-like plasma membrane domains likely contributes to the inability of the morphine-stimulated MOR to redistribute and therefore controls the sustained increase in cytosolic ERK activity. Knockdown of DSC1 (*green*) or JUP (*cyan*) facilitated a transient increase in nuclear ERK in response to morphine, indicative of a redistribution of the receptor at the plasma membrane (8).

The MOR is an important therapeutic target in the treatment of pain; however, the efficacy of current drugs is limited by their side effects (2). Different ligands that activate the MOR are able to cause distinct patterns of receptor phosphorylation, leading to differential receptor regulation and internalization (7). New drugs are currently being developed that exploit these key differences in receptor regulation following activation (29). Previously, we reported that different ligands also stimulate distinct spatiotemporal patterns of signaling (8). This occurred prior to MOR internalization, and it instead depended on the organization of the MOR at the plasma membrane. Here, using nonbiased proximity biotinylation MS, we have identified novel proteins that are in close proximity to the MOR following stimulation with DAMGO *versus* morphine. Although it is possible that the fusion of MOR with APEX2 may alter the proteins that can interact with the receptor, we were able to confirm that the identified protein networks are important for the WT MOR by using target knockdown or by directly measuring activation of signaling pathways. We find that DAMGO stimulation of MOR causes an activation of Rac1, dependent on IQGAP1 and CRKL scaffolding proteins (Fig. 5). Both scaffolding proteins are also required for DAMGO-mediated increases in nuclear ERK activity. In contrast, morphine increases the proximity between the MOR and desmosomal proteins (including DSC1 and JUP) (Fig. 5). Knockdown of DSC1 and JUP appears to release MOR from its restricted microdomain and allow transient increases in nuclear ERK in response to morphine.

The use of unbiased proximity biotinylation proteomics has allowed us to identify a novel signaling pathway for MOR; only DAMGO, but not morphine, causes the activation of the small Rho GTPase Rac1. Rho GTPases such as Rac1 modulate the actin cytoskeleton in many cells, including neurons (30), where the actin cytoskeleton is critical for the morphology of dendritic spines (31, 32). More than 90% of excitatory synaptic transmission occurs at dendritic spines (32), and they are critical for normal cognitive function and development (32). Interestingly, the function and morphology of dendritic spines are severely affected following chronic morphine treatment (33, 34). Chronic morphine treatment collapsed dendritic spines in hippocampal neuronal cultures, whereas treatment with DAMGO increased dendritic spine formation (35). As overexpression of Rac1 can induce the formation of dendritic spines (36), it is likely that selective activation of Rac1 by DAMGO, and not morphine, contributes to these morphological changes in neurons. We found that IQGAP1 was required for the DAMGO-mediated increase in Rac1 activity. Consistent with this, previous studies have shown that IQGAP1 can bind Rac1 (and maintain it in the active, GTP-bound form), the actin cytoskeleton, and is necessary for dendritic spine formation in hippocampal neurons (20, 37, 38). IQGAP1 is a large scaffolding protein that can interact with more than 90 proteins and is therefore proposed to act as both “a junction (integrating receptor signals) and a node (diversifying signals to multiple outputs)” (20, 38). In addition to integrating the signals of small GTPases (such as Rac1), IQGAP1 can constitutively bind other receptors, including the chemokine GPCRs, CXCR2 and CXCR4, and serves as a scaffold in the ERK-signaling cascade by binding directly to Raf, MEK1/2, and ERK1/2 (23, 38). This suggests that IQGAP1 could be a critical and specific scaffolding protein for MOR signaling in response to DAMGO but not morphine. In addition to IQGAP1, we found that the scaffolding protein CRKL was essential for increases in both Rac1 and ERK activity following DAMGO stimulation of the MOR. CRKL is an adaptor protein containing SH2 and SH3 protein–binding domains, allowing it to assemble protein complexes. CRK family adaptor proteins can increase the activity of Rac1 due to an SH3-domain interaction with the Rac1 GEFs, DOCK180 and Sos (39), and CRKL can increase ERK activity dependent on Rac1 (23). The identification of both CRKL and IQGAP1 as mediators of DAMGO-specific signaling of the MOR shows that distinct ligands induce differential assembly of extensive MOR–protein-interaction networks.

This concept is further supported by the unique protein-interaction networks that were identified following morphine stimulation of the MOR. Morphine stimulation of the MOR caused an enrichment of proteins implicated in the formation of desmosomes. Desmosomes are specialized regions of the plasma membrane. Although the principal function of desmosomes is adhesion and maintenance of tissue integrity, they may also act as signaling hubs (16). In fact, activation of the δ-opioid receptor in keratinocytes has been shown to reorganize desmosomes to promote cell detachment and migration (40). Three protein families constitute the core components of desmosomes: transmembrane cadherin proteins, including desmogleins and desmocollins (DSGs and DSCs), armadillo proteins (plakoglobin or JUP and plakophilins), and desmoplakin (41). Plakoglobin (junction plakoglobulin or JUP, γ-catenin) binds to both DSGs and DSCs to assemble desmosomes (42, 43). Morphine stimulation of MOR caused an enrichment of biotinylated JUP, DSC1, and DSG1. Knockdown of DSC1 and JUP desmosomal proteins allowed the morphine-stimulated MOR to cause a transient increase in nuclear ERK. We previously reported a similar transformation of the morphine spatiotemporal signaling profile to mimic that of DAMGO (*i.e.* transient nuclear ERK), following inhibition of Gβγ, PKC, or disruption of the plasma membrane architecture (8). Interestingly, PKCα regulates the dynamics of desmosome assembly: inhibition of PKC decreases desmosome assembly (44), the translocation of desmoplakin to the plasma membrane depends on PKC (28), and PKC activation promotes desmosome fluidity (45).

Desmosome assembly is a dynamic process involving clustering of DSC into nucleation sites, recruitment of DSG, followed by multiple phases of desmoplakin recruitment (46). Although we, and others, have shown endogenous expression of desmosome component proteins in HEK293 cells (28, 47), these cells do not form mature desmoplakin-containing desmosomes (48). Desmosomes are found mainly in the epithelia and heart; however, hybrid junctions, which contain components of both adherens junctions and desmosomes, have been reported in cardiomyocytes, vascular endothelial cells, and neurons (49, 50). Both desmosomes and adherens junctions are cadherin-based multiprotein complexes that couple cell–cell adhesion to the interfilament network (desmosomes) or the actin cytoskeleton (adherens junctions) (51). DSC and DSG have homology to the classical cadherins (*e.g.* E-cadherin and N-cadherin), and JUP (plakoglobin and γ-catenin) is highly homologous to β-catenin (51). In fact, exogenous expression of JUP can displace β-catenin from adherens junctions (52), and in JUP knockout mice, β-catenin is incorporated into desmosomes (53). Neuronal synapses are adherens junctions that are specialized for chemical transmission (54). In this setting cell-adhesion molecules, such as N-cadherin, maintain synaptic connections and can regulate the efficacy of synaptic transmission and plasticity (54, 55). JUP was associated with N-cadherin at all stages of synapse development in ciliary neurons (56) and, together with desmoplakin, was identified bound to N-cadherin in synapse-enriched lysate from hippocampal neurons (50). The authors proposed that the JUP/desmoplakin complex may tether cytoskeletal elements to a subset of synaptic junctions (50). Taken together, it seems clear that the desmosome-interaction network identified in HEK293 cells could also play an important role in MOR activation in neurons. Whether activation of MOR by morphine facilitates increased proximity of the receptor to classical desmosomes or a specialized hybrid junction (“desmosome-like platform”) is currently unknown. We propose that under normal conditions, morphine stimulation of the MOR activates a Gα_i/o_–Gβγ–PKC phosphorylation pathway that stabilizes the formation of a desmosome-like platform in close proximity to MOR. This restricts MOR movement and limits the receptor to cause a sustained increase in cytosolic ERK.

In conclusion, the use of APEX2 proximity biotinylation proteomics has allowed us to identify novel protein-interaction networks that are specific for DAMGO *versus* morphine stimulation of the MOR. This has provided mechanistic insights into previous observations of functional differences between these ligands. We found that DAMGO, but not morphine, can additionally activate Rac1, and we identified IQGAP1 and CRKL as essential scaffolds and integrators of DAMGO-mediated signaling. Additionally, the distinct spatiotemporal signaling profile of morphine appears to be dependent on a Gα_i/o_–Gβγ–PKCα stabilization of a desmosome-like platform. This may restrict the distribution of the MOR at the plasma membrane to control sustained cytosolic ERK signaling. Knockdown of desmosomal proteins DSC1 or JUP likely released the MOR from this plasma membrane domain to facilitate a transient increase in nuclear ERK. Future studies will need to evaluate the relevance of these protein interactions in more complex systems and *in vivo*. Identifying MOR-interaction networks that control differential spatiotemporal signaling represents an important step toward understanding how compartmentalized signaling contributes to the beneficial and clinically limiting effects of opioid analgesics.

## Experimental procedures

### Drugs

DAMGO was from Sigma and morphine HCl was from GlaxoSmithKline.

### Constructs

Mouse FLAG–MOR was from M. Christie (University of Sydney, Australia), and FLAG–MOR–APEX2 was from N. Veldhuis (Monash University, Australia). β-Arrestin 2-Venus was from K. Pfleger (Harry Perkins Institute of Medical Research, Australia); KRas-RLuc8 was from N. Lambert (Georgia Regents University), and GRK2 was from M. Smit (Vrije Universiteit Amsterdam, The Netherlands).

CAMYEL was from ATCC. The cytoEKAR EGFP–mRFP (Addgene plasmid 18680) and nucEKAR EGFP–mRFP (Addgene plasmid 18682) were from K. Svoboda (25). RaichuEV–Rac1 and Raichu–Cdc42/Cdc42CT (21, 22) were from M. Matsuda and K. Aoki (Kyoto University, Japan) and are contained in the pCAGGS vector (57) from J. Miyazaki (Osaka University, Japan).

### Antibodies

Immunostaining (confocal microscopy), immunoblotting, and in-cell Western blottings were performed using primary antibodies recognizing β-arrestin 1/2 (Cell Signaling Technology 46745; rabbit, 1:1000), β-actin (Cell Signaling Technology 3700S; mouse, 1:5000), α-tubulin (Cell Signaling Technology 3873;mouse, 1:4000), β-tubulin (Cell Signaling Technology 2146S; rabbit, 1:500), GRK2 (Santa Cruz Biotechnology sc-562; rabbit, 1:200), Rac1 (Millipore 05-389; mouse, 1:4000), DSC1 (Abcam ab150382; rabbit, 1:1000), DSG1 (Abcam ab124798; rabbit, 1:1000), IQGAP1 (Abcam ab86064; rabbit, 1:2000), JUP (Abcam ab184919; rabbit, 1:1000), CRKL (Abcam ab151791; rabbit, 1:1000), FLAG M2 for confocal microscopy (Sigma F3165; mouse, 1:1000), and FLAG M2 for in-cell Western blottings (Sigma F3165; mouse, 1:200).

Primary antibodies were detected using the following fluorescent secondary antibodies: goat anti-mouse 680 (LI-COR 926-68070; 1:10,000 for immunoblotting or 1:800 for in-cell Western blottings); goat anti-rabbit 800 (LI-COR 926-32211; 1:10,000 for immunoblotting or 1:800 for in-cell Western blottings); goat anti-mouse AlexaFluor 488 (ThermoFisher Scientific A28175; 1:500 for confocal microscopy). Endogenous Rac1 and α-tubulin (immunoblotting) were detected using a HRP-linked antibody (Cell Signaling Technology 7076; mouse, 1:4000).

### Cell culture and transfection

HEK293 cells (ATCC, negative for mycoplasma contamination) were grown in Dulbecco's modified Eagle's medium (DMEM) supplemented with 5% (v/v) fetal bovine serum (FBS). All assay dishes and plates were coated with poly-d-lysine (5 μg/cm^2^). HEK293 cells were transfected using linear polyethyleneimine (58).

### RNA-seq

RNA was extracted from two passages of HEK293 cells (P0 and P37) using the RNeasy mini kit (Qiagen), and transcriptome sequencing was performed by the Beijing Genomics Institute.

### Confirmation of target knockdown by siRNA

HEK293 cells in 6-well plates were transfected with 25 nm scrambled, IQGAP1, CRKL, DSC1, or JUP SMARTpool ON-TARGETplus siRNA (GE Dharmacon) for 48 h (quantitative RT-PCR) or 72 h (immunoblotting). Immunoblotting was used to confirm knockdown at the protein level for IQGAP1, CRKL, and JUP. DSC1 was not detectable by immunoblotting due to low expression in HEK293 cells (Fig. S8), so we used quantitative RT-PCR to confirm knockdown.

For confirmation of knockdown by immunoblotting, cells were lysed by resuspending in RIPA buffer (150 mm NaCl, 1% v/v Nonidet P-40, 0.5% w/v sodium deoxycholate, 0.1% w/v SDS, 50 mm Tris-Cl, pH 8.0; supplemented with 1 mm EDTA and protease mini EDTA-free inhibitor mixture (Roche Applied Science)) and incubating for 30 min on ice. The samples were sonicated on ice (30 s at 30% amplitude; Qsonica Q125) and centrifuged (10,000 × *g* for 10 min at 4 °C), and the supernatants were mixed with Laemmli sample buffer and incubated at 95 °C for 5 min. Samples were stored at −80 °C prior to immunoblotting.

For confirmation of knockdown by quantitative RT-PCR, RNA was extracted from HEK293 cells using the RNeasy mini kit (Qiagen). Quantitative RT-PCR was performed in duplicate from 500 ng of RNA. cDNA was produced using iScript Reverse Transcription Supermix (Bio-Rad) and 2720 Thermal Cycler (Applied Biosystems). Quantitative PCR used the TaqMan Fast Advanced Master Mix (Invitrogen) and a CFX384 Real-time System C1000 Touch (Bio-Rad). TaqMan probes (Applied Biosystems) used in this study were as follows: *DSC1* (Hs00245189_m1) and *HPRT1* (Hs02800695_m1). The 2^−Δ^*^Ct^* method (59) was used to analyze results, and data are expressed as 2^−Δ^*^Ct^* (difference in *C_t_* values of the gene of interest relative to the housekeeping gene, *HPRT1*) from six biological replicates.

### Measurement of endogenous Rac1 protein

HEK293 cells in 6-well plates were transfected with 25 nm scrambled, IQGAP1, or CRKL SMARTpool ON-TARGETplus siRNA (GE Dharmacon) for 72 h. Cells were lysed by resuspending in ice-cold buffer (150 mm NaCl, 10 mm MgCl_2_, 1% v/v Nonidet P-40, 0.1% w/v SDS, 50 mm Tris, pH 7.5, 1 mm phenylmethylsulfonyl fluoride, protease inhibitor mixture), incubating for 10 min on ice, then passing through a 21-gauge needle 30 times. The samples were centrifuged (400 × *g* for 3 min at 4 °C), and the protein in the supernatant was quantified using a Bradford protein assay, and supernatants were mixed with Laemmli sample buffer. Samples were stored at −80 °C prior to immunoblotting (50 μg total protein per lane).

### Immunoblotting: confirmation of protein knockdown

Proteins were resolved by SDS-PAGE using precast 4–15% Mini-PROTEAN TGX gels (Bio-Rad) and transferred to 0.45-mm low-fluorescence polyvinylidene difluoride membranes (Bio-Rad) using a Trans-Blot SD Semi-Dry Transfer Cell (for 75 min at 10 V; Bio-Rad). Membranes were blocked for 1 h at room temperature (5% w/v BSA in PBS with 0.1% v/v Tween 20 (PBS-T)) and incubated with primary antibody overnight at 4 °C (diluted in 1% w/v BSA). Membranes were washed, incubated with secondary antibody (diluted in PBS-T) for 1 h at room temperature, and washed. Immunoreactivity was detected by fluorescence using the Odyssey Classic IR Imager (LI-COR Biosciences), with resolution set at 169 μm.

### Immunoblotting: endogenous Rac1 protein

Proteins were resolved by SDS-PAGE using 15% gels and transferred to 0.45-μm nitrocellulose membranes (Amersham Biosciences) using a Mini Trans-Blot Electrophoretic Transfer Cell (for 1 h at 100 V; Bio-Rad). Membranes were blocked for 1 h at room temperature (5% w/v skim milk in Tris-buffered saline (TBS) with 0.2% v/v Tween 20 (TBS-T)) and incubated with primary antibody overnight at 4 °C (diluted in 1% w/v skim milk). Membranes were washed, incubated with secondary antibody (diluted in TBS-T) for 1 h at room temperature, and washed before a final wash in TBS. Immunoreactivity was detected by enhanced chemiluminescence using ECL Prime Western blotting System (GE Healthcare).

### In-cell Western assay

HEK293 cells were transfected with 55 ng/well FLAG–MOR or FLAG–MOR–APEX2 in black optically-clear 96-well plates. Following transfection, the cells were fixed with 4% (v/v) paraformaldehyde in PBS (20 min at room temperature) and then either left intact or permeabilized in 0.1% (v/v) Triton X-100 in PBS (three 10-min washes at room temperature). The cells were blocked (5% w/v BSA in PBS, with 0.1% v/v Tween 20 added to permeabilized samples) for 2 h at room temperature followed by overnight incubation with primary antibodies at 4 °C (diluted in 1% w/v BSA in PBS, with 0.1% v/v Tween 20 added to permeabilized samples). After washing (three 5-min washes with PBS, with 0.1% v/v Tween 20 added to permeabilized samples), the cells were incubated with secondary antibodies for 1 h in the dark at room temperature (diluted in 1% w/v BSA in PBS, with 0.5% v/v Tween 20 added to permeabilized samples). The cells were washed (three 5-min washes with PBS at room temperature, with 0.1% v/v Tween 20 added to permeabilized samples), the buffer was aspirated, and the dry plate was scanned using the Odyssey Classic IR Imager (LI-COR Biosciences), with resolution set at 169 μm and offset at 3 mm.

### Confocal imaging

Cells expressing FLAG–MOR or FLAG–MOR–APEX2 were fixed with 4% (w/v) paraformaldehyde in PBS for 20 min at 4 °C and then were washed three times for 5 min with PBS. Cells were blocked in PBS containing 3% (v/v) normal goat serum and 0.1% (w/v) saponin for 1 h at room temperature and then were incubated with primary antibody in PBS containing 1% (v/v) normal horse serum and 0.1% (w/v) saponin overnight at 4 °C. Cells were washed three times for 5 min with PBS and incubated with secondary antibody in PBS containing 1% (v/v) normal horse serum and 0.1% (w/v) saponin for 2 h at room temperature. The cells were washed three times for 5 min in PBS before the addition of 1 μg/ml 4′,6-diamidino-2-phenylindole nuclear stain for 5 min at room temperature in the dark. Cells were washed twice for 5 min with PBS. To visualize the localization of FLAG–MOR or FLAG–MOR–APEX2, cells were observed with a Leica SP8 confocal microscope and HCX PL APO 63Å∼ CS2 (NA, 1.40) oil objective.

### BRET assays: cAMP and β-arrestin recruitment

HEK293 cells in 10-cm dishes were co-transfected with 2 μg of FLAG–MOR or FLAG–MOR–APEX2 and 1 μg of CAMYEL (BRET cAMP biosensor). For β-arrestin recruitment assays, HEK293 cells in 10-cm dishes were co-transfected with 1 μg of FLAG–MOR or FLAG–MOR–APEX2, 1 μg of KRas-RLuc8, 2 μg of GRK2, and 4 μg of β-arrestin 2-Venus. After 24 h, the cells were re-plated in 96-well white opaque plates (CulturPlate, PerkinElmer Life Sciences). Forty eight hours after transfection, the medium was removed, and cells were washed once with Hanks' balanced salt solution (HBSS, Gibco), before incubation of the cells in HBSS for 30 min at 37 °C. For cAMP assays, the cells were incubated with 5 μm coelenterazine h (NanoLight) in the dark for 10 min at 37 °C, before co-addition of 30 μm forskolin (to activate adenylyl cyclase) and vehicle (0.1% v/v DMSO) or increasing concentrations of DAMGO for 5 min at 37 °C. β-Arrestin recruitment assays measured the proximity of β-arrestin 2-Venus to KRas-RLuc8 (a marker of the plasma membrane), which allowed direct comparison of the effects of activation of MOR *versus* MOR–APEX2 in the same assay system. Co-expression of GRK2 ensured a detectable β-arrestin 2-Venus/KRas-RLuc8 BRET signal (7, 60). For β-arrestin recruitment assays, 5 μm coelenterazine h was added to the cells, and the BRET baseline was measured every minute for 15 min, before addition of vehicle (0.1% v/v DMSO) or 1 μm DAMGO with BRET measurements continued every 1 min for 20 min. BRET was measured using a LUMIstar OMEGA plate reader (BMG Labtech) with sequential integration of the signals detected at 475 ± 30 and 535 ± 30 nm with filters with the appropriate band pass. Data are shown as the BRET ratio (calculated as the ratio of the YFP signal to the RLuc signal) expressed as the ligand-induced change in BRET compared with 30 μm forskolin alone for the cAMP assay or as the 20 min area under the curve (AUC) following baseline correction for the β-arrestin recruitment assay.

### Spatial ERK and Rac1 using high-content ratiometric FRET imaging

Ratiometric FRET imaging was performed as described previously (8, 10). We detected changes in ERK using EKAR targeted to the cytosol or the nucleus, which undergoes a conformational change after ERK phosphorylation of a target sequence (25). Changes in Rac1 and Cdc42 activity were detected using RaichuEV–Rac1 or Raichu–Cdc42, respectively, which undergo a conformational change after GTP displaces GDP within a target sequence (21, 22).

HEK293 cells were seeded in black, optically clear 96-well plates and grown to 70% confluency before co-transfection with FLAG–MOR or FLAG–MOR–APEX2 (55 ng per well) and a FRET biosensor (40 ng per well). For experiments with siRNA, HEK293 cells were co-transfected with an additional 25 nm scrambled, CRKL, IQGAP1, JUP, or DSC1 SMARTpool ON-TARGETplus siRNA (GE Dharmacon) for 72 h. Before the experiment, HEK293 cells were partially serum-restricted overnight in 0.5% (v/v) FBS/DMEM. Fluorescence imaging was performed using a high-content GE Healthcare INCell 2000 Analyzer with a Nikon Plan Fluor ELWD ×40 (NA, 0.6) objective and FRET module as described previously (58). For CFP/YFP (RaichuEV–Rac1 and Raichu–Cdc42) emission ratio analysis, cells were sequentially excited using a CFP filter (430/24 nm) with emission measured using YFP (535/30 nm) and CFP (470/24 nm) filters and a polychroic filter optimized for the CFP/YFP filter pair (Quad3). For GFP/RFP (cytoEKAR and nucEKAR) emission ratio analysis, cells were sequentially excited using a fluorescein isothiocyanate (FITC) filter (490/20 nm) with emission measured using dsRed (605/52 nm) and FITC (525/36 nm) filters and a polychroic filter optimized for the FITC/dsRed filter pair (Quad4). For control experiments (Fig. 1 and Fig. S2), cells were imaged every 1 min for 20 min (image capture of 14 wells per min); for experiments using siRNA, cells were imaged every 1.5 min for 21 min (image capture of 18 wells per min). At the end of each experiment, the same cells were stimulated with the following positive controls to maximally activate the biosensor: 200 nm phorbol 12,13-dibutyrate for EKAR or a mixture of 1 μm isoprenaline, 50 ng/ml EGF, 10 μm AlCl_3_, and 10 mm NaF for RaichuEV–Rac1 and Raichu–Cdc42 (58). Only HEK293 cells with >5% change in *F*/*F*_0_ (FRET ratio relative to baseline for each cell) after stimulation with positive controls were selected for analysis, and the data were expressed relative to the positive control (*F/F*_max_). The average *F/F*_max_ was calculated for each experiment and combined. Data were analyzed using in-house scripts written for the FIJI distribution of ImageJ (61), as described previously (58).

### APEX2 proximity biotinylation: sample preparation

For analysis by immunoblotting, HEK293 cells in 3.5-mm dishes were transfected with 1 μg of FLAG–MOR–APEX2. For analysis by MS, HEK293 cells in 10-cm dishes were transfected with 5 μg of FLAG–MOR–APEX2, with three plates used for each experimental condition. 48 h post-transfection, the media were replaced with that containing 500 μm biotin-tyramide (Iris Biotech GmbH) and incubated for 1 h. During this time, the cells were treated with either vehicle (0.1% v/v DMSO), 1 μm DAMGO, or 1 μm morphine for the indicated time. Biotin labeling was initiated by addition of 1 mm H_2_O_2_ for 1 min. The media were removed; the cells were placed on ice, washed three times with ice-cold quenching buffer (10 mm sodium ascorbate, 5 mm Trolox, 10 mm sodium azide in PBS), and then incubated in quenching buffer for 20 min. The quenching buffer was removed, and the cells were scraped in lysis buffer (50 mm Tris-HCl, pH 7.4, 500 mm NaCl, 0.2% w/v SDS, protease mini EDTA-free inhibitor mixture (Roche Applied Science), 1 mm DTT). The lysates were transferred to conical tubes containing pre-chilled 20% (v/v) Triton X-100 in 50 mm Tris-HCl, pH 7.4. The samples were sonicated on ice (five 30-s on/off cycles at 50% amplitude; Qsonica Q125) and centrifuged (16,000 × *g* for 10 min at 4 °C), and the supernatants were incubated with either 25 μl (immunoblotting) or 100 μl (MS) of streptavidin magnetic beads (ThermoFisher Scientific) overnight at 4 °C.

After magnetic separation (3 min at room temperature), the supernatant was removed, and beads were washed with wash buffer 1 (2% w/v SDS) for 8 min at room temperature. The samples were again separated on a magnetic stand (3 min at room temperature) and then washed once with wash buffer 2 (0.1% w/v deoxycholic acid, 1% v/v Triton X-100, 1 mm EDTA, 500 mm NaCl, 50 mm HEPES, pH 7.5) followed by wash buffer 3 (0.5% w/v deoxycholic acid, 0.5% v/v Nonidet P-40, 1 mm EDTA, 250 mm LiCl, 10 mm Tris-HCl, pH 7.4) and then final two washes with 100 mm HEPES, pH 8.1. For immunoblotting, the proteins were eluted in Laemmli buffer by boiling for 15 min. For MS, the proteins were eluted in FASP lysis buffer (100 mm Tris, pH 7.6, 4% w/v SDS, 100 mm DTT) at 95 °C for 15 min. The supernatant was digested with trypsin using the FASP protein digestion kit (Expedeon) overnight at 37 °C. Digested peptides were desalted using ZipTips (Merck Millipore). Samples were eluted (70% v/v acetonitrile, 0.1% v/v formic acid) and then dried by SpeedVac (Labconco). Samples were resuspended (2% v/v acetonitrile, 1% v/v formic acid, 33 nm iRT peptides) by sonication at 37 °C for 10 min before LC-MS/MS.

### APEX2 proximity biotinylation: experimental design and statistical rationale

HEK293 cells were transfected with MOR–APEX2 and stimulated with the indicated treatments (vehicle, DAMGO, and morphine) for 10 or 60 min on four independent occasions (four biological replicates, six samples in each replicate). Time-matched vehicle-treated samples were used as controls. Peptide and protein searches were performed using MaxQuant at a FDR threshold of 1% (described in detail below). The fourth replicate was removed from further analysis due to a consistently low number of proteins identified across all experimental conditions. All 374 identified proteins were input for Ingenuity Pathway Analysis (described in detail below). The statistical significance of the ligand-induced change in log_2_ for proteins of interest was determined by two-way ANOVA with Dunnett's multiple comparison test.

### APEX2 proximity biotinylation: LC-MS/MS

Using a Dionex UltiMate 3000 RSLCnano system equipped with a Dionex UltiMate 3000 RS autosampler, an Acclaim PepMap RSLC analytical column (75 μm × 50 cm, nanoViper, C18, 2 μm, 100 Å; Thermo Fisher Scientific), and an Acclaim PepMap 100 trap column (100 μm × 2 cm, nanoViper, C18, 5 μm, 100 Å; Thermo Fisher Scientific), the tryptic peptides were separated by increasing concentrations of 80% acetonitrile, 0.1% formic acid at a flow of 250 nl/min for 120 min and analyzed with a QExactive Plus mass spectrometer (Thermo Fisher Scientific) operated in data-dependent acquisition mode using in-house, LFQ-optimized parameters.

In detail, the eluent was nebulized and ionized using a nano electrospray source with a distal coated fused silica emitter (New Objective). The capillary voltage was set at 1.7 kV. The Q Exactive mass spectrometer was operated in the data-dependent acquisition mode to automatically switch between full MS scans and subsequent MS/MS acquisitions. Survey full-scan MS spectra (*m*/*z* 375–1575) were acquired in the Orbitrap with 70,000 resolution (at *m*/*z* 200) after accumulation of ions to a 3 × 10^6^ target value with a maximum injection time of 54 ms. Dynamic exclusion was set to 15 s. The 12 most intense multiply charged ions (*z* ≥2) were sequentially isolated and fragmented in the collision cell by higher-energy collisional dissociation with a fixed injection time of 54 ms, 17,500 resolution, and automatic gain control target of 2 × 10^5^.

### APEX2 proximity biotinylation: peptide and protein identification

Acquired .raw files were analyzed with MaxQuant version 1.6.0.16 (62) to identify and quantify peptides and proteins using the human SwissProt database downloaded from Uniprot in November, 2017. The database, which contained 20,243 entries, was appended by the iRT peptide sequences. The parameters for MaxQuant searches were as follows: precursor mass tolerance was set to 20 ppm (parts per million) for the first search and 4.5 ppm for the main search. Carbamidomethylation of cysteines was entered as a fixed modification, whereas oxidation of methionines and acetylation of protein N termini were set as variable modifications. Trypsin was used as the enzymatic protease, and a maximum of two missed cleavages was allowed. The FDR for peptide and protein identification was set to 1%, using the target-decoy approach, and only unmodified and razor peptides were used for quantification. Label-free quantification was switched on with minimum peptide ratio count of 2. The ProteinGroups.txt file generated by MaxQuant was further analyzed with Perseus version 1.6.0.7 (63) after proteins that were identified “only-by-site,” reverse hits, and potential contaminants were removed.

### APEX2 proximity biotinylation: IPA

IPA (version 01–14) (64) requires values in all the experimental conditions input for analysis. To maximize the proteins included in this analysis, we performed two rounds of imputation to generate values where a signal was not detected for all experimental conditions.

First, a fixed log_2_ value of 16 was imputed for the missing experimental conditions when a protein was not identified in vehicle-treated samples but was found in samples treated with either DAMGO or morphine or both, and vice versa. The value used was smaller than the lowest log_2_ value identified from all 374 proteins (log_2_ of 17.1). This allowed us to keep proteins in the dataset that are either recruited to MOR or move away from MOR following stimulation. The second imputation step was for missing values for proteins that were not detected in every biological replicate.

In this case, the missing values were subjected to several rounds of imputation with Perseus version 1.6.0.7 to define the optimal imputation parameters. The missing values were randomly replaced from a normal distribution by varying the parameters for width (of the Gaussian distribution relative to the standard deviation of measured values) and down shift (of the Gaussian distribution used for the random numbers). It is important that the imputed values meet three criteria: they do not form a separate normal distribution; they start at approximately the same point for all biological replicates; and the distribution is narrower than that of the measured values. We found a 0.5 width and 1.5 downshift were the best-fit parameters across all replicates.

All values (experimental and imputed) from the three biological replicates were expressed as average log_2_ change compared with vehicle-treated controls. These values were imported into IPA for analysis. First, a core analysis was performed to quickly identify relationships, mechanisms, functions, and pathways specific to a particular treatment condition (*i.e.* DAMGO 10 or 60 min and morphine 10 or 60 min). Second, a comparison between the four treatment conditions was performed to allow prediction of activation/inhibition of canonical pathways and generation of networks of interacting proteins. This allowed us to generate protein-interaction network heat maps (Figs. 2*B*, 3*A,* and 4*A* and Tables 1–3). Statistical analysis was performed without imputed values (Fig. 2*C*, 3*C*, and 4*C*).

## Author contributions

S. C., C. H., B. L., A. B. G., R. B. S., A. M. E., and M. L. H. formal analysis; S. C., C. H., B. L., E. A. M., A. B. G., and M. L. H. investigation; S. C., and M. L. H. methodology; S. C., A. B. G., R. B. S., A. M. E., M. C., and M. L. H. writing-review and editing; R. B. S., A. M. E., M. C., and M. L. H. supervision; M. C. and M. L. H. conceptualization; M. C. and M. L. H. funding acquisition; M. L. H. resources; M. L. H. writing-original draft; M. L. H. project administration.

## Supplementary Material

Supporting Information

## Footnotes

3 The abbreviations used are:

MOR: μ-opioid receptor
APEX2: ascorbate peroxidase from the soy bean
BRET: bioluminescence resonance energy transfer
CRKL: Crk-like protein
DAMGO: [d-Ala^2^,*N*-MePhe^4^,Gly-ol] enkephalin
DSC1: desmocolin-1
DSG1: desmoglein-1
ERK: extracellular signal-regulated kinase
FRET: Förster resonance energy transfer
GPCR: G-protein-coupled receptor
GRK2: G-protein receptor kinase 2
IPA: Ingenuity Pathway Analysis software
IQGAP1: IQ motif containing GTPase-activating protein 1
JUP: junction plakoglobin
PKC: protein kinase C
Rac1: Ras-related C3 botulinum toxin substrate 1
AUC: area under the curve
ANOVA: analysis of variance
SH: Src homology
DMEM: Dulbecco's modified Eagle's medium
FBS: fetal bovine serum
CFP: cyan fluorescent protein
FDR: false discovery rate
EGFP: enhanced GFP
RFP: red fluorescent protein
FITC: fluorescein isothiocyanate.
