# Supplementary material for: Ligand-dependent spatiotemporal signaling profiles of the μ-opioid receptor are controlled by distinct protein-interaction networks
Source: J Biol Chem. 2019 Sep 12;294(44):16198–213. doi: 10.1074/jbc.RA119.008685 (PMC6827304; doi:10.1074/jbc.RA119.008685)
Supplement: Supporting Information [file supp_294_44_16198__index.html]

Ligand-dependent spatiotemporal signaling profiles of the mu-opioid receptor are controlled by distinct protein-interaction networks — MOR-interaction networks control spatiotemporal signaling — Ligand-dependent spatiotemporal signaling profiles of the μ-opioid receptor are controlled by distinct protein-interaction networks — MOR-interaction networks control spatiotemporal signaling — Supporting Information 

# Ligand-dependent spatiotemporal signaling profiles of the μ-opioid receptor are controlled by distinct protein-interaction networks

## Supporting Information

- Supporting Information - Figures - Supporting Figures
- Supporting Information Table S1 - Proteins identified and quantified by LC-MS/MS
