# Supplementary material for: Ligand-dependent spatiotemporal signaling profiles of the μ-opioid receptor are controlled by distinct protein-interaction networks
Source: J Biol Chem. 2019 Sep 12;294(44):16198–213. doi: 10.1074/jbc.RA119.008685 (PMC6827304; doi:10.1074/jbc.RA119.008685)
Supplement: Supporting Information [file supp_RA119.008685_144637_1_supp_385340_pxf18k.pdf]

Supporting Information

Ligand-dependent spatiotemporal signaling profiles of the mu-opioid receptor are controlled by distinct protein-protein interaction networks

Srgjan Civciristov, Cheng Huang, Bonan Liu, Elsa A. Marquez, Arisbel B. Gondin, Ralf B. Schittenhelm, Andrew M. Ellisdon, Meritxell Canals, and Michelle L. Halls

Figure S1. Validation of MOR-APEX vs MOR wild-type.
Figure S2. Spatiotemporal analysis of ERK activity following stimulation of MOR.
Figure S3. Hierarchical clustering heat map for activation of canonical signaling pathways.
Figure S4. Analysis of Cdc42 activity following stimulation of MOR.
Figure S5. Effect of knockdown of CRKL or IQGAP1 on endogenous Rac1 protein levels.
Figure S6. Analysis of nuclear ERK activity following stimulation of MOR.
Figure S7. Effect of knockdown of CRKL, IQGAP1, DSC1 or JUP on the maximal FRET change displayed by RaichuEV-Rac1 and nucEKAR.
Figure S8. HEK293 cells express low levels of DSC1 and DSG1.

Table S1. Protein identification following streptavidin pulldown and LC-MS/MS.

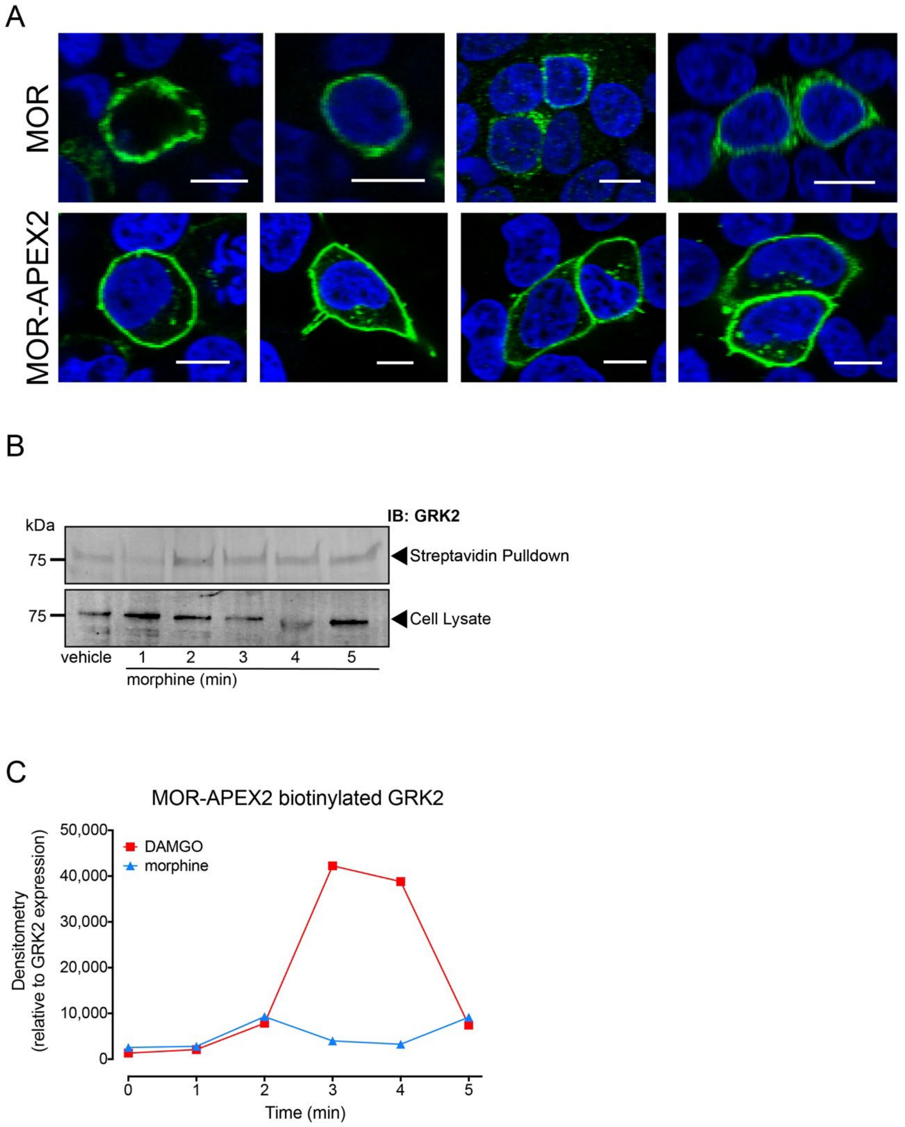

Figure S1. Validation of MOR-APEX vs MOR wild-type. A. Representative images confirming the localization of MOR (upper panels) and MOR-APEX2 (lower panels) at the plasma membrane of HEK293 cells detected by immunostaining of the N-terminal FLAG-tag and confocal microscopy. FLAG-tag immunostaining is shown in green, and the nucleus (DAPI stain) is shown in blue. Scale bar: 10 µm. B. Analysis of the proximity of MOR-APEX2 to transiently expressed GRK2 in HEK293 cells following stimulation with 1 µM morphine, streptavidin pulldown and immunoblotting. C. Quantification of data in B and Figure 1I. The densitometry of the GRK2 band from streptavidin pulldown is expressed relative to the amount in the cell lysate at each time point.

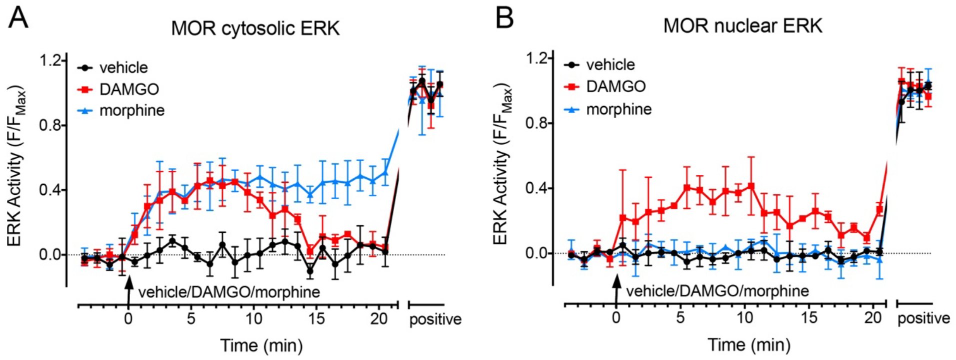

Figure S2. Spatiotemporal analysis of ERK activity following stimulation of MOR. Analysis of the spatial activation of ERK in HEK293 cells transiently expressing MOR and stimulated with vehicle, 10 nM DAMGO or 100 nM morphine. A. Analysis of cytosolic ERK activity using cytoEKAR (n=3). B. Analysis of nuclear ERK activity using nucEKAR (n=3). Symbols represent means and error bars indicate standard deviation of the mean from 3 experiments.

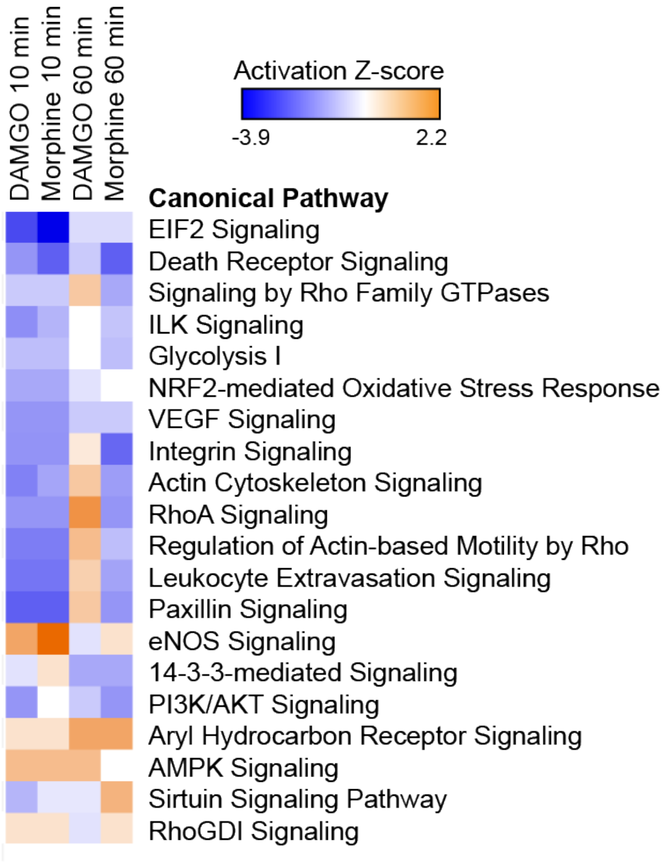

Figure S3. Hierarchical clustering heat map for activation of canonical signaling pathways. Analysis of proximity biotinylation data using Ingenuity Pathway Analysis (IPA) software allowed hierarchical clustering of the canonical signaling pathways that were predicted to be activated following stimulation of HEK293 cells expressing FLAG-MOR-APEX2 with 1 μM DAMGO or 1 μM morphine for 10 min or 60 min compared to a time-matched vehicle control. The heat map shows activation or inhibition of the signaling pathways expressed as a Z-score.

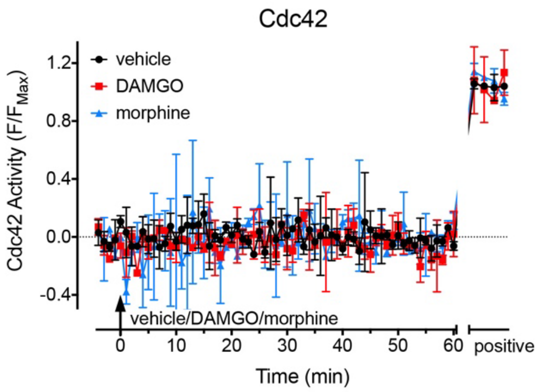

Figure S4. Analysis of Cdc42 activity following stimulation of MOR. Analysis of the activation of Cdc42 using Raichu-Cdc42 in HEK293 cells transiently expressing MOR and stimulated with vehicle, 1 µM DAMGO or 1 µM morphine (n=2). Symbols represent means and error bars indicate standard deviation of the mean from 2 experiments.

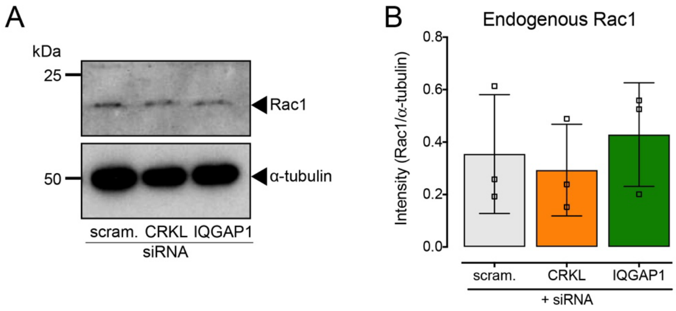

Figure S5. Effect of knockdown of CRKL and IQGAP1 on endogenous Rac1 protein levels. The amount of endogenous Rac1 protein in HEK293 cell lysates co-transfected with MOR and scrambled siRNA, CRKL siRNA or IQGAP1 siRNA was assessed. A. Representative immunoblot. B. Grouped intensity measurements from 3 independent experiments. Scatter plots show individual data points, bars represent means and error bars indicate standard deviation of the mean from 3 experiments.

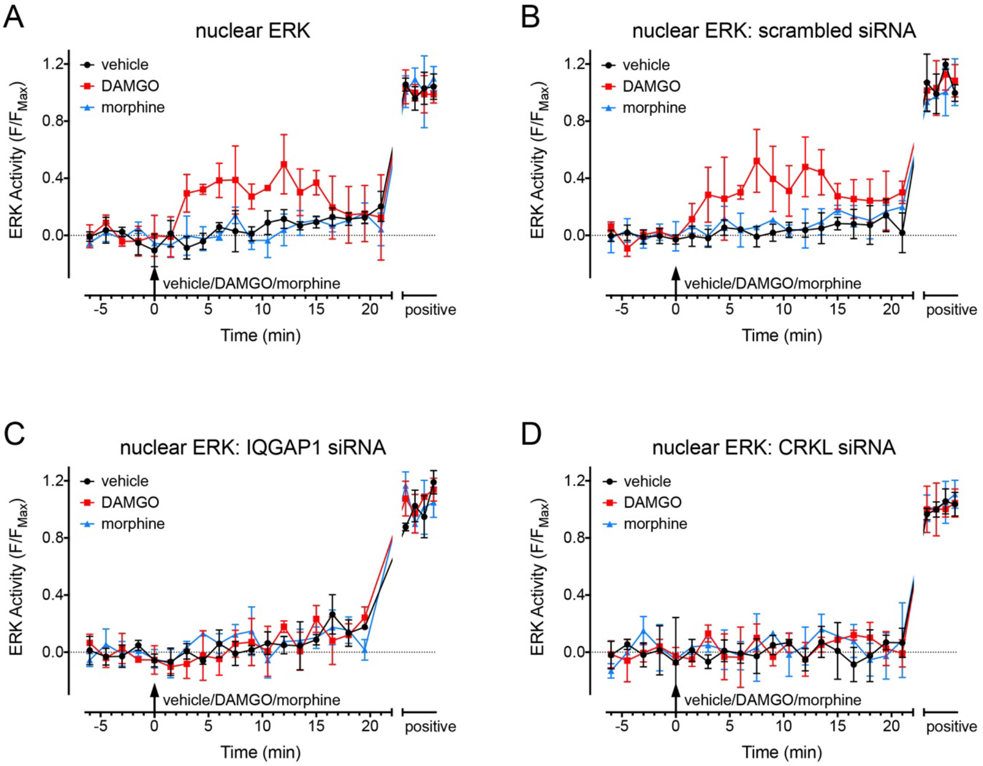

Figure S6. Analysis of nuclear ERK activity following stimulation of MOR. Analysis of the activation of nuclear ERK using nucEKAR in HEK293 cells transiently expressing MOR and stimulated with vehicle, 1 µM DAMGO or 1 µM morphine. A. Control cells (n=3). B. Cells transfected with scrambled siRNA (n=3). C. Cells transfected with IQGAP1 siRNA (n=3). D. Cells transfected with CRKL siRNA (n=3). Symbols represent means and error bars indicate standard deviation of the mean from 3 experiments.

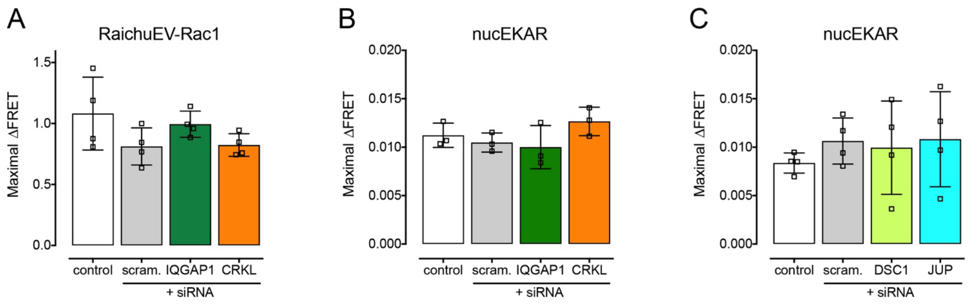

Figure S7. Effect of knockdown of CRKL, IQGAP1, DSC1 or JUP on the maximal FRET change displayed by RaichuEV-Rac1 and nucEKAR. Analysis of the maximal FRET change displayed by RaichuEV-Rac1 or nucEKAR in HEK293 cells transiently expressing MOR and stimulated with the relevant positive control. A. Maximal RaichuEV-Rac1 FRET change induced by addition of a cocktail of 1 μM isoprenaline, 50 ng/ml EGF, 10 μM $AlCl_3$, and 10 mM NaF, in cells co-transfected with pcDNA (control), scrambled siRNA, IQGAP1 siRNA or CRKL siRNA (n=4). B. Maximal nucEKAR FRET change induced by addition of 200 nM PDBu in cells co-transfected with pcDNA (control), scrambled siRNA, IQGAP1 siRNA or CRKL siRNA (n=3). C. Maximal nucEKAR FRET change induced by addition of 200 nM PDBu in cells co-transfected with pcDNA (control), scrambled siRNA, DSC1 siRNA or JUP siRNA (n=4). Scatter plots show individual data points, bars represent means and error bars indicate standard deviation of the mean from n experiments as stated.

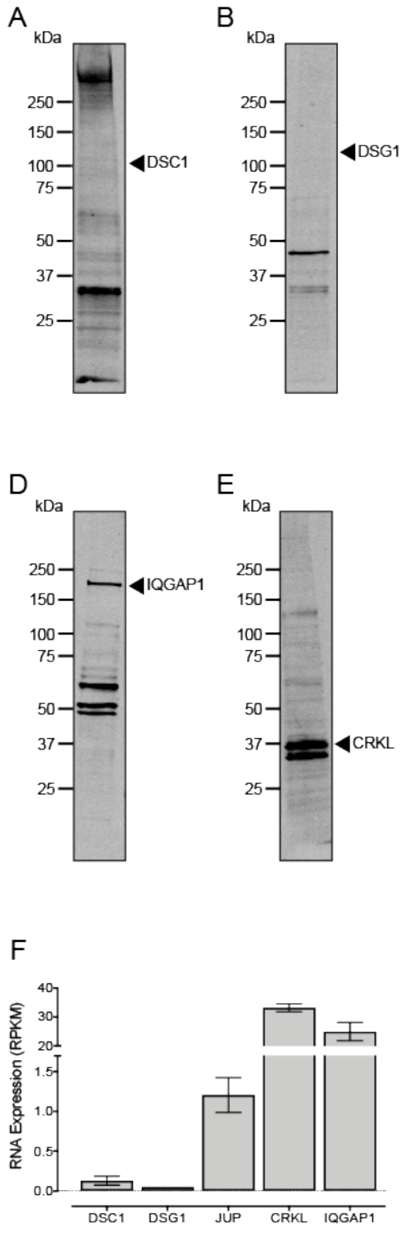

Figure S8. HEK293 cells express low levels of DSC1 and DSG1. In order to confirm knockdown of desmosomal proteins, DSC1, DSG1 and JUP, and the scaffolding proteins, IQGAP1 and CRKL, we attempted to detect endogenous protein levels in HEK293 cells using immunoblotting. A-B. We were unable to detect bands at the appropriate molecular weight using antibodies against A. DSC1 or B. DSG1. C-E. We detected endogenous levels of proteins by immunoblotting using antibodies against C. JUP, D. IQGAP1 and E. CRKL. F. RNA-sequencing data from our HEK293 cells showed very low levels of DSC1 and DSG1, but higher levels of JUP, CRKL and IQGAP1 (all three of which we could detect at the protein level using immunoblotting). Bars represent means, and error bars show the standard deviation from the mean of 2 experiments.
